# Probiotic formulations and gastro-intestinal diseases in the paediatric population: a narrative review

**DOI:** 10.1097/MS9.0000000000002007

**Published:** 2024-04-04

**Authors:** Amna Siddiqui, Ramsha Haider, Syeda Ilsa Aaqil, Laiba Imran Vohra, Khulud Qamar, Areesha Jawed, Nabeela Fatima, Alishba Adnan, Vidhi Parikh, Sidhant Ochani, Md. Al Hasibuzzaman

**Affiliations:** a Department of Medicine, Karachi Medical and Dental College; b Department of Medicine, Jinnah Sindh Medical University; c Department of Medicine, Ziauddin Medical University; d Department of Medicine, Dow University of Health and Sciences, Karachi; e Department of Medicine, Khairpur Medical College, Khairpur Mir’s, Pakistan; f Parul Institute of Medical Sciences and Research, Parul University, Vadodara; g Mentor, International Society of Chronic Illnesses, India; h Institute of Nutrition and Food Sciences, University of Dhaka, Dhaka, Bangladesh

**Keywords:** adolescent, children, gut, microbiome signature, microbiota, synbiotic

## Abstract

**Background/Aim:**

Probiotics are live microbial supplements that improve the microbial balance in the host animal when administered in adequate amounts. They play an important role in relieving symptoms of many diseases associated with gastrointestinal tract, for example, in necrotizing enterocolitis (NEC), antibiotic-associated diarrhea, relapsing Clostridium difficile colitis, Helicobacter pylori infections, and inflammatory bowel disease (IBD). In this narrative review, the authors aim to evaluate the role of different probiotic formulations in treating gastrointestinal diseases in pediatric population aged 18 years or younger and highlight the main considerations for selecting probiotic formulations for use in this population.

**Methodology:**

The authors searched PubMed and Clinicaltrials.gov from inception to 24th July 2022, without any restrictions. Using an iterative process, the authors subsequently added papers through hand-searching citations contained within retrieved articles and relevant systematic reviews and meta-analyses.

**Results:**

The effectiveness of single-organism and composite probiotics in treating gastrointestinal disorders in pediatric patients aged 18 or under were analyzed and compared in this study. A total of 39 studies were reviewed and categorized based on positive and negative outcomes, and compared with a placebo, resulting in 25 studies for single-organism and 14 studies for composite probiotics. Gastrointestinal disorders studied included NEC, acute gastroenteritis (AGE), Acute Diarrhea, Ulcerative Colitis (UC), and others. The results show that probiotics are effective in treating various gastrointestinal disorders in children under 18, with single-organism probiotics demonstrating significant positive outcomes in most studies, and composite probiotics showing positive outcomes in all studies analyzed, with a low incidence of negative outcomes for both types.

**Conclusion:**

This study concludes that single-organism and composite probiotics are effective complementary therapies for treating gastrointestinal disorders in the pediatric population. Hence, healthcare professionals should consider using probiotics in standard treatment regimens, and educating guardians can enhance the benefits of probiotic therapy. Further research is recommended to identify the optimal strains and dosages for specific conditions and demographics. The integration of probiotics in clinical practice and ongoing research can contribute to reducing the incidence and severity of gastrointestinal disorders in pediatric patients.

## Graphical abstract

**Figure:**
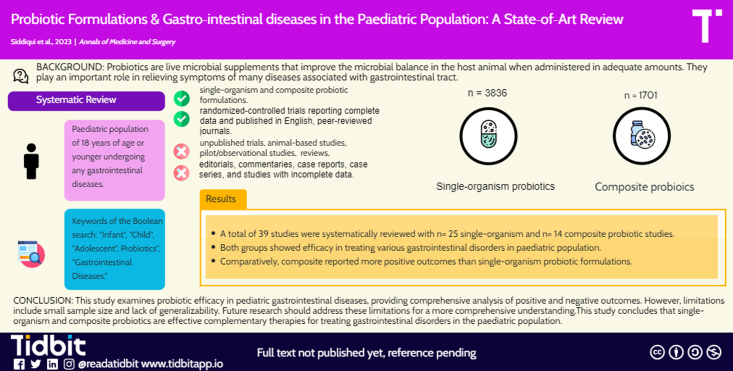

## Introduction

Highlights

- Probiotics are dietary supplements and foods consisting of yeast and bacteria that are commonly used, especially the Lactobacillus and Bifidobacterium species. The diseases related to the gastrointestinal tract (GIT) are often caused by an imbalance in the microbiota found in GIT.
- Probiotics are believed to play a crucial role in relieving GIT-related disease symptoms and beneficially regulating the microbiota composition. The study supports the role of different probiotic formulations in treating GI diseases among individuals aged 18 years or younger.
- A comprehensive search was conducted in PubMed and Clinicaltrials.gov from inception to 24th July 2022, without any restrictions. The search included iterative processes and hand-searching citations contained within retrieved articles, systematic reviews, and meta-analyses.
- The study includes a descriptive and comparative analysis between single-organism and composite probiotics. Results demonstrate that probiotics are effective in treating various GI disorders, including NEC, FAP, AGE, Acute Diarrhea, Ulcerative Colitis, and many others, and have been compiled into 39 studies categorized by title and outcomes.
- The positive outcomes highlight the drug’s effectiveness in improving health, while the negative outcome explains any adverse effects that the drug may have shown. The study emphasizes the importance of gaining further insights into various gut microbes and microbiomes with specific demographic to reduce GI disorders and strengthen the gut.

Functional digestive problems have become more prevalent in recent years and can be associated with various conditions such as gastrointestinal cancers, intestinal obstruction, ulcers, and reflux diseases. Functional gastrointestinal diseases (FGIDs) are the most common type of gastrointestinal disorders, especially in infants, children, and adolescents. As of 2021, around 40% of the world’s population suffers from some form of gastrointestinal disease^1^. The occurrence rates of FGIDs in pediatric populations have been documented to range from 27 to 40.5% among infants and toddlers aged 0 to 3 years, and from 9.9 to 27.5% among children and adolescents aged 4–18 years^2^.

Dysbiosis has been linked to various metabolic and chronic medical conditions including gastrointestinal disorders. However, the patterns of dysbiosis have been inconsistently observed across different countries and life stages^3^. As the human gut microbiome changes significantly over a lifetime, age-specific differences may provide insight into microbiome-mediated effects on health^4^. The systematic review by Abdukhakimova *et al*.^5^ found no clear microbiota signature associated with celiac disease in children’s fecal and/or duodenal samples due to heterogeneity in study design, but suggests that certain fecal microbiota elements, particularly Bifidobacterium spp., such as Bifidobacterium longum, may be useful as diagnostic/prognostic biomarkers and for probiotic therapy and require further investigation. Similarly, study by Poddighe and Kushugulova^6^, suggested that although a clear microbiome signature for celiac disease has not been identified in humans, several studies suggest that the gut microbiota can impact disease onset and progression through various mechanisms^7^. Clinical studies on the salivary microbiome in celiac disease are less common but may provide better correlation with the duodenal bacterial environment. Further studies on the salivary microbiome in different populations are necessary to explore its usefulness in understanding celiac disease pathogenesis with potential clinical implications.

Probiotics play an important role in relieving symptoms of many other diseases associated with gastrointestinal tract for example in, necrotizing enterocolitis (NEC), antibiotic-associated diarrhea, relapsing Clostridium difficile colitis, Helicobacter pylori infections, and inflammatory bowel disease (IBD). When it pertains to extremely preterm infants between the ages of 2 weeks and 2 months, NEC is the most prevalent major gastrointestinal condition and the leading cause of mortality. The condition mostly affects babies born before 32 weeks of pregnancy, and its frequency is inversely correlated with gestational age^8^. It is linked to the use of antibiotics, acid suppression, enteral diluted hydrochloric acid, and enteral antibiotics, all of which change the microbiome of the infant’s gut. These findings lend credence to the idea that dysbiosis, or aberrant gut flora, is a primary cause of NEC^8^. The hallmarks of NEC include acute inflammation, penetration of gas into the portal venous system and bowel wall, ischemic necrosis of the intestinal mucosa, and invasion by enteric gas-forming organisms. These symptoms have been linked to a higher risk of neurodevelopmental (ND) impairment (NDI)^9^. As far as antibiotic associated diarrhea is concerned, of individuals who obtain antibiotics, up to 35% suffer from diarrhea^10^. This is attributed to the gut flora’s colonization resistance^11^. It is frequently mild, but it can occasionally be severe and even fatal, particularly when Clostridium difficile infection (CDI) is present^10^. In addition to frequent watery bowel movements, urgency, and dyspepsia, is linked to changes in intestinal microbiota, mucosal integrity, and vitamin/mineral metabolism. If severe, it can cause toxic megacolon, pseudomembranous colitis, electrolyte imbalances, diminution in volume, and, very rarely, death^11^. Dysbiosis is also linked to inflammatory bowel disease (IBD), which includes Crohn’s disease and ulcerative colitis. Disproportionate immune responses against commensal intestinal bacteria have been observed in IBD patients, and these responses may be essential in driving intestinal inflammation^12,13^. Others, however, speculate that the main cause of inflammation in IBD is an imbalanced gut flora^14^. Commensal enteric microbes can lead to the following complications in IBD patients; superinfection with intestinal pathogens can trigger flare-ups of the illness; and opportunistic infections become more significant when immunosuppressive medication is used widely. In addition to hepatocellular abscesses, sepsis, and endocarditis, secondary bacterial invasion of mucosal ulcers also frequently results in common septic local complications such as abscesses and fistulae^14^.

According to the International Scientific Association for Probiotics and Prebiotics (ISAPP) consensus statement on the scope and appropriate use of the term probiotic and prebiotics; probiotics are live microbial feed supplements that beneficially affect the host animal by improving microbial balance when administered in adequate amounts, while prebiotics are nondigestible dietary compounds that stimulate the growth and activity of specific bacterial populations^15,16^. To ensure safety and effectiveness, a set of established criteria has been developed for selecting probiotics, as summarized in Table 1 ^17^. Following this, there has been a recent increase in global consumption of nonprescription probiotics for improving overall health, as they have been found to regulate the composition of the microbiota by promoting beneficial bacteria and inhibiting harmful bacteria. However, conflicting clinical findings exist regarding the effectiveness of many probiotic strains and preparations.

**Table 1:** Criteria for use as a probiotic^17^.

• The organism being utilized must be recognized, that is, its genus, species, and strain must be known
• The organism must be deemed viable to consume: *not infectious or harboring genes for antibiotic resistance *not converting to bile acids or degrading to the intestinal mucosa
• It needs to endure intestinal transit: tolerance of bile and acid
• It has to stick to the mucosa and colonize the gut (at least for a short period)
• It must have known and documented impacts on health: *synthesize antimicrobials and combat harmful germs *a minimum of one phase 2 research demonstrating a benefit
• During storage and processing, it must remain stable

Therefore, to address the variability in probiotics research in terms of disparity of studied strains this systematic review aims to assess the efficacy of different probiotic formulations, at the strain level, in treating gastrointestinal diseases in the pediatric population. It provides a comprehensive review of current evidence on the use of probiotics as a complementary therapy for treating gastrointestinal disorders in pediatric patients, including the effectiveness of single-organism and composite probiotic formulations, ongoing clinical trials, and recommendations for healthcare professionals and guardians. The ultimate goal of this review is to assess the positive and negative outcomes of utilizing probiotic formulation and improve the management of gastrointestinal disorders in pediatric patients and promote further research in this field.

## Methodology

### Data sources AND search strategy

We searched PubMed and Clinicaltrials.gov from inception to 24th July 2022, without any restrictions. In PubMed, two search strategies were combined, that is, S1 and S2 (Table 2) searches included Medical Subject Headings [MeSH] and limits to title and abstract [Title/Abstract]. S2 was added to exclude studies limited to animals or involving both animal and human participants. Using an iterative process, we subsequently added papers through hand-searching citations contained within retrieved articles and relevant systematic reviews and meta-analyses.

**Table 2:** Detailed search strategy.

S1: ((infant*[Title/Abstract] OR baby[Title/Abstract] OR babies[Title/Abstract] OR newborn*[Title/Abstract] OR neonat*[Title/Abstract] OR neo nat*[Title/Abstract] OR child*[Title/Abstract] OR toddler*[Title/Abstract] OR adolescen*[Title/Abstract] OR teen*[Title/Abstract] OR teenager*[Title/Abstract] OR youth[Title/Abstract] OR juvenile*[Title/Abstract] OR “Infant”[Mesh] OR “Child”[Mesh] OR “Adolescent”[Mesh])) AND (Probiotics[Title/Abstract] OR “Probiotics”[Mesh]) AND (“Gastrointestinal Diseases”[Mesh])) NOT S2: ((“Animals”[Mesh]) NOT (“Animals”[Mesh] AND “Humans”[Mesh]))

Inclusion criteria: The studies needed to provide complete data related to the study topic, be randomized controlled trials, focus on evaluating the efficacy of various probiotic formulations in patients with gastrointestinal-related diseases, and be published in peer-reviewed journals in English. We specifically sought studies involving pediatric populations aged 18 years or younger diagnosed with a range of gastrointestinal diseases. Additionally, we included studies that investigated both single-organism and composite probiotic formulations, regardless of dosage or form. In determining the relevance of study outcomes, we considered only those reported in studies where the *P*-value was less than or equal to 0.05, indicating statistical significance. Furthermore, to facilitate analysis, we classified study outcomes as either positive, indicating health enhancement, or negative, suggesting potential harmful effects of the intervention. These classifications are detailed comprehensively in Table 3 for clarity and reference.

**Table 3:** List of randomized controlled trials analyzing different types of that is, single-organism and composite probiotic formulations.

Single-organism probiotic vs. placebo						
	Study and patient characteristics					
Study	Study location	Sample size (*n*= number of individuals)	Study population (Gastrointestinal disease)	Intervention (Probiotic Formulation)	Positive outcome	Negative outcome
Benor S. *et al*.^11^	Israel	58	NEC	*L reuteri* DSM 17938 (1 108 Colony-Forming Units/D)	Intervention might decrease the incidence of NEC in breastfed infants	N/A
Romano C. *et al*.^11^	Sicily	60	Functional Abdominal Pain (FAP)	*Lactobacillus reuteri*	The intervention reduced perceived abdominal pain intensity	N/A
Serce O. *et al*.^18^	Turkey	208	NEC	*Saccharomyces boulardii*	N/A	The intervention did not decrease the incidence of NEC or sepsis
Demirel G. *et al*.^19^	Turkey	271	NEC	*S. boulardii*	Feeding intolerance and clinical sepsis were found to be significantly lower in the probiotic group	The intervention was not effective at reducing the incidence of death or NEC in very low birth weight (VLBW) infants
Pieścik-Lech M. *et al*.^20^	Poland	88	AGE	LGG and smectite versus LGG alone	LGG plus smectite and LGG alone are equally effective for treating young children with AGE. The combined use of the two interventions is not justified	N/A
Francavilla R. *et al*.^21^	Italy	74	Acute Diarrhea	*Lactobacillus reuteri* DSM 17938 derived from *L. reuteri* ATCC 55730	The intervention was found beneficial in reducing the frequency, duration and recrudescence rate of the disease	N/A
Oliva S. *et al*.^22^	Italy	40	Mild to Moderate Ulcerative Colitis (UC)	*L. reuteri*	The intervention was effective in improving mucosal inflammation and changing mucosal expression levels of some cytokines involved in the mechanisms of inflammatory bowel disease	N/A
Maldonado J. *et al*.^23^	Spain	215	Incidence Of Infections	*Lactobacillus fermentum* CECT5716 *(L. Fermentum)*	The intervention was found useful for the prevention of community-acquired gastrointestinal and upper respiratory infections	N/A
Sari FN. *et al*.^24^	Turkey	221	NEC	*Lactobacillus sporogenes*	Feeding intolerance was significantly lower in the probiotics group than in the control group	The intervention showed no significant difference in the incidence of death or NEC between the groups
Dinleyici EC. *et al*.^25^	Turkey	68	Blastocystis Hominis Infection	*Saccharomyces boulardii*	Metronidazole or S. boulardii has potential beneficial effects on B. hominis infection	N/A
Indrio F. *et al*.^26^	Italy	42	Regurgitation	*Lactobacillus reuteri*	Intervention reduces gastric distension and accelerates gastric emptying. In addition, this probiotic strain seems to diminish the frequency of regurgitation	N/A
Francavilla R. *et al*.^12^	Italy	141	Irritable Bowel Syndrome (IBS) or (FAP)	LGG	The intervention seemed to significantly reduce the frequency and severity of abdominal pain in children with IBS; this effect is sustained and may be secondary to the improvement of the gut barrier	N/A
Martens U. *et al*.^13^	Iran	52	IBS	LGG	The key IBS symptoms (abdominal pain, stool frequency), as well as the other symptoms (bloating, mucous and blood in stool, need for straining at stools, urge to defecate), improved significantly during treatment. Global assessment of therapy by parents and doctors was altogether positive	No adverse effects were shown
Coccorullo P. *et al*.^14^	Naples	44	Functional Chronic Constipation	*Lactobacillus reuteri* (DSM 17938)	The intervention caused a higher frequency of bowel movements	The intervention showed no improvement in stool consistency and episodes of inconsolable crying episodes
Hojsak I. *et al*.^15^	Croatia	742	Nosocomial Gastrointestinal Tract Infections	LGG	The intervention caused the risk for gastrointestinal infections, vomiting episodes and diarrheal episodes, episodes of gastrointestinal infections, episodes of respiratory tract infections that lasted 3 days to significantly decrease	N/A
Hojsak I. *et al*.^16^	Daycare centers are located in 4 separate locations in the Zagreb area	281	Gastrointestinal Tract Infections	LGG	Intervention reduced the risk of gastrointestinal infections, vomiting episodes, and diarrheal episodes. However, intervention caused no reduction in the number of days with gastrointestinal symptoms	N/A
Baldassarre ME. *et al*.^17^	Bari hospital	30	Hematochezia and Fecal Calprotectin	LGG	LGG resulted in significant improvement of hematochezia and fecal calprotectin compared with the extensively hydrolyzed casein formula (EHCF) alone	N/A
Sentongo TA. *et al*.^27^	Chicago, IL	21	Short Bowel Syndrome (SBS)	LGG	N/A	Findings do not support empiric LGG therapy to enhance IP in children with SBS
Szajewska H. *et al*.^28^	Poland	29	Rectal Bleeding	LGG	N/A	The intervention was ineffective in treating rectal bleeding in breastfed infants. No adverse effects were reported
Bauserman M. *et al*.^29^	Children’s medical center pediatric gastroenterology	50	IBS	LGG	The intervention showed improvement in abdominal distention	Lactobacillus GG was not superior to placebo in the treatment of abdominal pain in children with IBS
Sýkora J. *et al*.^30^	Czech republic	86	Helicobacter Pylori	*Lactobacillus casei (L. casei)* DN-114 001	Eradication success was higher due to intervention	Side effects were infrequent
Dani C. *et al*.^31^	Italy	585	Urinary Tract Infection, Bacterial Sepsis and NEC	LGG	It was found that infants who received Lactobacillus GG were less affected by NEC after 1 week of treatment	The intervention was not effective in reducing the incidence of UTIs, NEC and sepsis in preterm infants
Saran S. *et al*.^32^	India	100	Diarrhea	*Lactobacillus acidophilus*	There were significantly fewer cases of diarrhea and fever due to the intervention	N/A
Rosenfeldt V. *et al*.^33^	Denmark	43	Acute Diarrhea	*L. reuteri* DSM 17938	The intervention was effective in reducing the duration of diarrhea	N/A
Guandalini S. *et al*.^34^	Eleven centers in 10 countries	287	Acute Diarrhea	LGG	The intervention was deemed safe and results were obtained in a shorter duration of diarrhea. There was less chance of a protracted course, and patients were discharged earlier from the hospital	No adverse effects (rash, drug-related fever or nausea, etc.) related to the synbiotic use were noted
Composite Probiotic vs. Placebo						
Muhammed Majeed. *et al*.^35^	Three clinical sites i) Mysore Medical College and K R Hospital, Mysore, India ii) Sapthagiri Institute of Medical Sciences and Research Center, Bangalore, India and iii) Kempegowda Institute of Medical Sciences, Bangalore, India	36	Diarrhea Predominant IBS	*Bifidobacterium breve, Lactobacillus casei* and *Galactooligosaccharides*	The intervention caused a significant change/decrease in clinical symptoms like bloating, vomiting, diarrhea, and abdominal pain. Stool frequency disease severity also decreased and the quality of life increased in the patient due to the intervention	No serious adverse effects were shown
Evette Van Niekerk. *et al*.^36^	Neonatal high care unit of Tygerberg Children’s Hospital (TBCH), Cape Town, South Africa	184	NEC	Probiotic Mixture (*Bifidobacteria infantis, Streptococcus thermophilus,* and Bifidobacteria bifidus; Solgar, Israel)	reduced the incidence of NEC reduction in the severity of disease was found in the HIV-exposed study group	The intervention failed to show that probiotics lowered the incidence of NEC in HIV-exposed premature infants
Ali İşlek. *et al*.^37^	Pediatric Emergency and Pediatric Gastroenterology Departments of the Akdeniz University Hospital	156	Acute Infectious Diarrhea	Infloran	The duration of diarrhea was significantly shorter in the synbiotic group than in the placebo The duration of diarrhea was shorter for patients who started the synbiotic therapy within the first 24 h than for those who started their treatment later	No adverse effects were shown
Xiaolin Wang. *et al*.^38^	Three medical centers—the Department of Pediatric Surgery, Tongji Hospital, Tongji Medical College, Huazhong University of Science and Technology; The First Hospital of Harbin Medical University; and Anhui Provincial Hospital	60	Hirschsprung’s Disease-Associated Enterocolitis (HAEC)	*Lactobacillus plantarum 299* and *Bifidobacterium infantis cure 21*	The incidence of HAEC (three out of 30, 10.0%) in the probiotic-treated group was significantly reduced the severity of HAEC in the probiotic-treated group was significantly reduced Probiotics-balanced T lymphocyte, IFN-γ, and IL-6 were significantly decreased inflammatory cytokine IL-10 was remarkably increased	N/A
Marta Olivares. *et al*.^39^	Hospital Universitari Sant Joan (Reus, Tarragona) and Hospital Universitario Sant Joan de Deu (Barcelona)	33	Colic Disease	a capsule containing either *B. longum* CECT 7347 (109 colony forming units)	The intervention caused a significant increase in the height percentile, decreased levels of peripheral CD3+ T lymphocytes and slightly reduced TNF-a concentration, The B. longum CECT 7347 group also had reduced numbers of the Bacteroides fragilis group and lower sIgA content in stools compared to the placebo group	No adverse events were reported during the intervention
Fernández-Carrocera LA. *et al*.^40^	Mexico	150	NEC	*Bifidobacterium infantis*, *Streptococcus thermophilus*, and *Bifidobacterium lactis* containing 1 × 10(9) Total Organisms)	The intervention caused a reduction of NEC frequency, significantly lowered risk for the combined risk of NEC or death	No adverse effects were shown during hospitalization
Braga TD. *et al*.^41^	Northeast brazil	231	NEC	VSL#3	The intervention reduced the occurrence of NEC (Bell’s stage ≥ 2). It was considered that an improvement in intestinal motility might have contributed to this result	N/A
Cazzola M. *et al*.^42^	France	135	Prevention of common winter diseases in children	*Lactobacillus acidophilus, Lactobacillus rhamnosus, Bifidobacterium bifidum, Bifidobacterium longum, Enterococcus faecium,*	Intervention decreased the risk of occurrence of common infectious diseases in children and limited the risk of school day loss	N/A
Guandalini S. *et al*.^43^	Italy (4) and in India (1) Chicago, IL (1)	59	IBS	*Bifidobacterium breve and Lactobacillus casei*	The intervention caused improvement in IBS symptoms	No adverse event was recorded
Lin HC. *et al*.^44^	Taiwan	217	NEC	Mixture of *Bifidobacterium longum* (BB536) and *Lactobacillus johnsonii* (La1 )	The incidence of death or NEC (stage 2) was significantly lower	No adverse effect, such as sepsis, flatulence, or diarrhea, was noted
Bin-Nun A. *et al*.^45^	Shaare zedek medical center	145	NEC	Probiotic Mixture (*Bifidobacteria infantis, Bifidobacteria bifidum, Bifidobacteria longum* and *Lactobacillus acidophilus*	reduced both the incidence and severity of NEC	No adverse effects were shown
Kliegman RM. *et al*.^46^	Houston, texas	155	NEC	*Bifidobacterium bifidum* and *Lactobacillus acidophilus*	The incidence of death or NEC (stage 2) was significantly lower	N/A
Vandenplas Y. *et al*.^47^	Belgium	111	Acute Diarrhea	Synbiotic food supplement Probiotical (*Streptoccoccus thermophilus, Lactobacillus rhamnosus, Lactobacillus acidophilus, Bifidobacterium lactis, Bifidobacterium infantis, fructo-oligosaccharides)*	The median duration of diarrhea was significantly 1 day shorter in the synbiotic than in the placebo group, associated with decreased prescription of additional medications	N/A
Miele E. *et al*.^48^	Italy	29	UC	VSL#3	Endoscopic and histological scores were significantly lower in the VSL#3 group Remission was achieved in 13 patients	No adverse effects were shown

Exclusion criteria: All the unpublished trials, animal-based studies, study designs, that is, pilot and observational studies, reviews, editorials, commentaries, case reports, case series, and studies reporting incomplete data were excluded.

## Results

### Data selection

**Figure 1:**
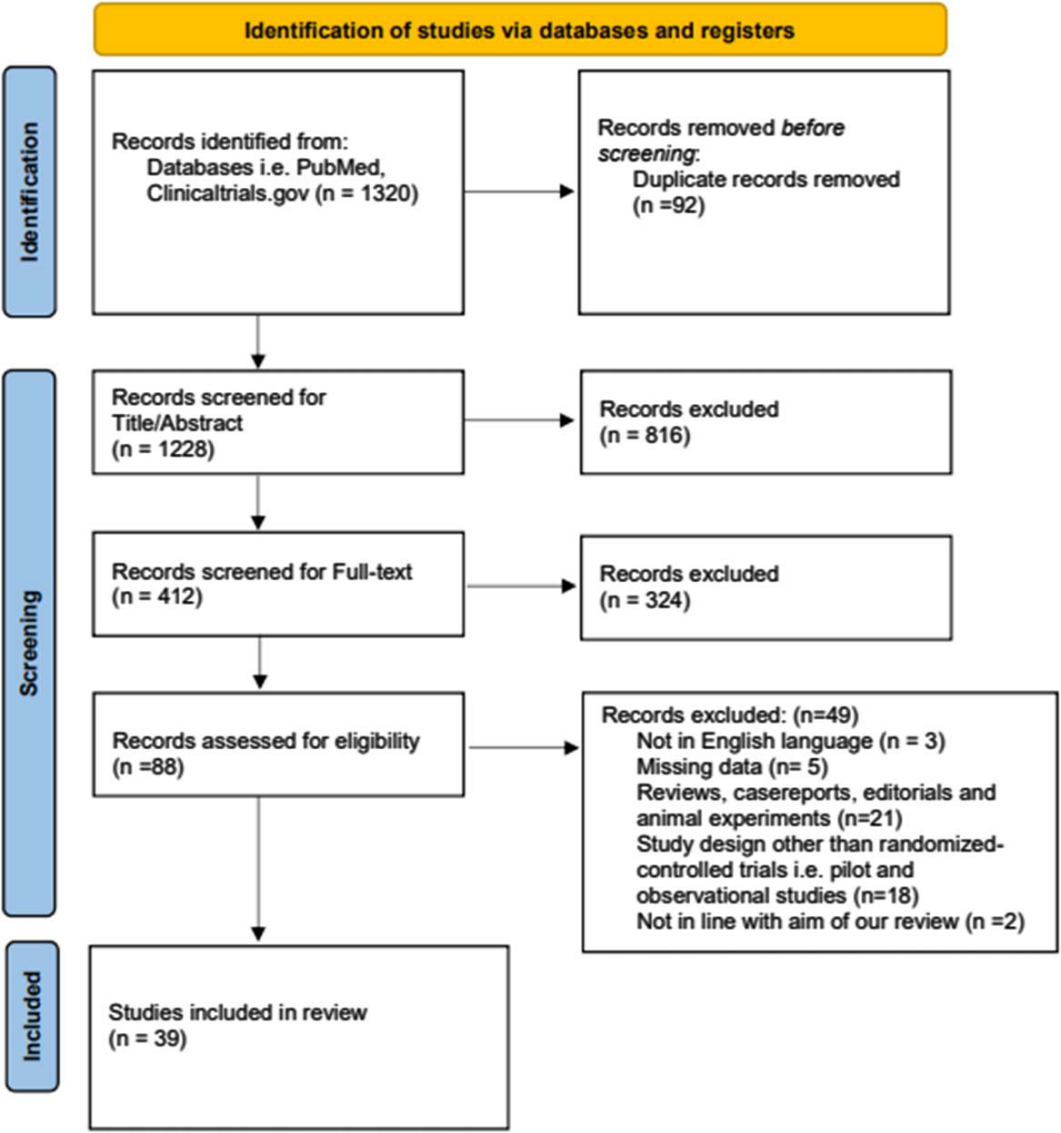
PRISMA flowchart summarising the screening process.

Following the primary search and after removing duplicates, we screened 1228 articles for relevance based on title and abstract and full-text. We also manually searched for additional articles by looking through reference lists of the included full-text. In the end, a total of 39 studies were included in our review, details of the screening process are displayed in the flowchart below (Fig. 1). A list of selected examples of probiotic formulations is provided in Table 4. The studies are summarized in Table 3 based on their characteristics and findings.

**Table 4:** Selected examples of probiotic formulations.

Single-organism probiotic	Composite probiotic
*Saccharomyces boulardii*	Ecologic®Relief: *Bifidobacteria (B.) bifidum, B. infantis, B. longum, Lactobacilli (L.) casei, L. plantarum and L. rhamnosus*
*Lactobacillus acidophilus*	A mixture of *Bifidobacterium breve, Lactobacillus casei* and *Galactooligosaccharid*es Probiotic Mixture *(Bifidobacteria infantis, Streptococcus thermophilus, and Bifidobacteria bifidus*)
*Bifidobacterium longum CECT 7347*	Infloran: *Lactobacillus acidophilus* and *Bifidobacterium infantis*
*Lactobacillus fermentum CECT5716*	Mixture of *Lactobacillus plantarum 299* and *Bifidobacterium infantis cure 21*
*Lactobacillus sporogenes*	Mixture of *Lactobacillus rhamnosu*s *GG* and *Bifidobacterium infantis*
*Lactobacillus rhamnosus GG* (LGG)	Mixture of oral bifidobacterium, lactobacillus acidophilus, and enterococcus Triple Viable Capsules
*Lactobacillus reuteri* (DSM 17938)	*Bifidobacterium infantis, Streptococcus thermophilus,* and *Bifidobacterium lactis,*
*Lactobacillus casei* (DN-114 001)	Mixture of *Lactobacillus acidophilus* and *Bifidobacteria lactis*
*Lactobacillus GG* (Dicoflor)	VSL#3 is composed of four strains of lactobacillus, three strains of bifidobacterium, and one strain of *Streptococcus salivarius subsp. Thermophiles*
	Mixture of *Lactobacillus acidophilus, Lactobacillus rhamnosus, Bifidobacterium bifidum, Bifidobacterium longum, Enterococcus faecium*
	Mixture of *Bifidobacterium breve* and *Lactobacillus casei*
	Mixture of *Bifidobacterium longum* (BB536) and *Lactobacillus johnsonii* (La1 )
	Probiotic mixture of *Bifidobacteria infantis, Bifidobacteria bifidum, Bifidobacteria longum and Lactobacillus acidophilus*
	Mixture of *Bifidobacterium bifidum* and *Lactobacillus acidophilus*
	Mixture of *L. acidophilus* and *B. infantis*

### Single probiotic

According to Table 3, of the 25 articles assessed, 22 reported at least one statistically significant positive outcome between single-organism probiotics vs placebo^18–21,23–37,45,46^. The remaining three reported no statistically significant positive outcome attributed to single-organism probiotics^38–40^. A study reported that single-organism probiotics decreased the incidence of necrotizing enterocolitis (NEC) in breastfed infants^17,37^. Another study reported that intervention reduced the intensity of abdominal pain^18,26,27,30^. Feeding intolerance and clinical sepsis were found to be significantly lower in the probiotic group according to another study^23,28^. Pieścik-Lech *et al*.^20^ reported that the combined use of Lactobacillus rhamnosus GG (LGG) plus smectite or LGG alone are effective in treating young children with acute gastroenteritis (AGE). Single-organism probiotics were found to be beneficial in decreasing the frequency and duration of the gastrointestinal diseases like that is, AGE, acute diarrhea, and NEC^20,32–34^. Additionally, it also improved mucosal inflammation and changed mucosal expression levels of some cytokines involved in the mechanism of inflammatory bowel disease^21^. These probiotics were found to result in more frequent bowel movements, decreased stomach bloating, and accelerated gastric emptying^25,28^. Hojsak *et al*. reported a decreased risk of respiratory tract infections and gastrointestinal infections with probiotics in children in daycare centers and pediatric facilities^35,36^.

In contrast, of the of the 25 articles assessed, 16 reported no statistically significant negative outcomes between single-organism probiotics vs placebo^18,20,21,23,25–27,29,30,32–35,37,41,45^. The remaining nine reported at least one statistically significant negative outcome attributed to single-organism probiotics^19,24,28,31,35,38–40,46^.

Few studies reported that single-organism probiotics did not decrease the incidence of NEC^19,24,31,38^. Additionally, no improvement in stool consistency was seen, but accompanied episodes of inconsolable crying were reported^28^. A study showed that intervention was ineffective in treating rectal bleeding in breastfed infants^40^. Lactobacillus rhamnosus therapy in children with short bowel syndrome did not improve intestinal permeability and was associated with conversion to positive hydrogen breath test results^46^.

### Composite probiotics

Of the 14 articles using composite probiotics assessed, all reported at least one statistically significant positive outcome between composite probiotics vs placebo^22,42–44,47–55^. One study reported that composite probiotics decreased clinical symptoms like bloating, vomiting, diarrhea, and abdominal pain^42,44^. It also decreased disease severity, consequently improving the quality of life in the patient^42,43^. Studies showed a lower incidence of NEC frequency with intervention in the HIV-exposed study group, preterm infants and low-birth neonates^43,44,47,49,53,54,56^. Taking composite probiotics within 24 h significantly decreased the duration of diarrhea compared to those who took it later^50^. Xiaolin *et al*.^51^ reported that T lymphocytes, IFN-γ, and IL-6 decreased, whereas IL-10 increased in patients treated with probiotics along with a decreased incidence of HAEC (3/30, 10%). Another study reported a decreased risk of common infectious diseases in children with probiotics, leading to a lower risk of school day loss^55^. Similarly, one study reported that the median duration of diarrhea was 1 day shorter in synbiotic food supplement Probiotical (Streptoccoccus thermophilus, Lactobacillus rhamnosus, Lactobacillus acidophilus, Bifidobacterium lactis, Bifidobacterium infantis, fructo-oligosaccharides) than the placebo group and hence, was associated with a decreased prescription of additional medications^48^. Another study reported that remission was achieved in active UC patients and endoscopic and histological scores were significantly lower in the VSL #3 group (Table 4) compared to the placebo group^22^.

In contrast, of from the 14 articles assessed, 13 reported no statistically significant negative outcomes between composite probiotics vs placebo^22,42,44,47,48,50–55,57^. The remaining reported one statistically significant negative outcome attributed to composite probiotics, the study reported that probiotics failed to decrease the incidence of NEC in HIV-exposed premature infants^43^.

### Discussion: a way forward

In this study, the effectiveness of single-organism and composite probiotics in treating gastrointestinal disorders in the pediatric population aged 18 or under were analyzed and compared. A total of 39 studies were reviewed and categorized based on their outcomes, which included positive and negative effects of the probiotics. The studies were compared with a placebo, both individually and as a group, resulting in 25 studies for single-organism probiotics and 14 studies for composite probiotics. Gastrointestinal disorders studied included NEC, AGE, Acute Diarrhea, UC, and others. The findings of the study emphasize the effectiveness of probiotics in treating various gastrointestinal disorders in pediatric aged 18 or younger, with single-organism probiotics showing significant positive outcomes in most studies and composite probiotics showing positive outcomes in all studies analyzed, with low incidence of negative outcomes for both types of probiotics.

The possible mechanism of activity of some probiotic characteristics may be present in a uniform manner across different species or even genera^58^. As such, efficacy may vary across different strains. For instance, both Bifidobacterium spp. and Lactobacillus spp. can generate the enzyme β-galactosidase, which can help address lactase insufficiency^59^. In contrast, other traits may be specific to certain species^60^ or even strains^61^, or may require interactions between different probiotic strains^62^. Some probiotic preparations, particularly those containing specific strains such as S. boulardii and LGG, have been shown in several meta-analyses and systematic reviews to help alleviate acute diarrhea in children and reduce its duration of diarrhea^63–65^. However, one recent updated meta-analysis based on large-scale randomized placebo-controlled trials^66,67^ involving over nine-tenth children in their total sample size with AGE concluded that large trials with low risk of bias suggest that probiotics are unlikely to have a significant impact on the incidence of diarrhea lasting 48 h or longer, and there is uncertainty regarding their effectiveness in reducing the duration of diarrhea^63^. Earlier guidelines that supported the use of probiotics in the management of AGE, based on lower-quality evidence, are now contradicting with these new findings.

There is evidence to suggest that probiotics may be effective in averting neonatal late-onset sepsis and NEC, a digestive illness that frequently impacts premature infants^68^. Research conducted on animals and human cell cultures proposes that specific types of probiotics, including LGG, could safeguard against NEC by strengthening the body’s defense mechanisms against harmful microorganisms, encouraging the growth of the immune system and cells lining the intestine, and reducing inflammation^69^. However, other trials involving very preterm infants in England using Bifidobacterium breve BBG-001 showed no significant effect on NEC or sepsis prevention^70^. Previous systematic reviews and meta-analyses have produced conflicting outcomes on the effectiveness of enteral probiotics in preterm infants. A 2014 Cochrane review involving more than 5000 infants concluded that the administration of enteral probiotics containing either Lactobacillus alone or in combination with Bifidobacterium can decrease the occurrence of NEC and mortality in preterm infants, but not nosocomial sepsis^71^. Similarly, different systematic review and meta-analysis showed that probiotics are useful in reducing the occurrence of late-onset sepsis in preterm infants when given as mixtures and exclusively to those fed with human milk^72^. However, other meta-analyses did not find any significant effect of probiotics in preventing NEC or sepsis in infants with extremely low birth weights^73,74^. Depending on the disease and the particular probiotic being used, there may also be variations among the ideal dosage and period of probiotic treatment. Any probiotic strain has to reach its ideal mass or dosage for it to thrive and colonize the gut. Present literature shows that probiotics must be living and at high enough dosages (usually 106–107 colony-forming units (cfu)/g of product) in order to be effective^75^. However, Stool colonization rates indicate large studies often employ doses of 1×108 or 1×109^76^, and greater doses (1×1010) do not appear to enhance colonization rates^77^.

Prebiotics, on the other hand, which comprise different combinations of acidity, fructo-oligosaccharides, and galacto-oligosaccharides from nonhuman milk, have been researched in preterm children for a long time. These prebiotic mixes promote gastric peristalsis, minimize eating intolerance, raise stool sIgA, modify the fecal microbiota, and lower stool pH levels^78^. In contrast to probiotics which have some evidence of reducing incidence of NEC and sepsis in preterm infants, a meta-analysis of seven prebiotics placebo-controlled randomized clinical studies reveals that they play no role in the reduction in NEC, sepsis, or death in preterm infants^79^.

To the best of our knowledge, the present study is the first one to examine the efficacy of probiotics on multiple gastrointestinal diseases among pediatric population younger or 18 years of age, highlighting all positive and negative outcomes for each probiotic to enable a more detailed comparison of the specific strains of each probiotic when used individually or in a composite. This approach provides valuable insights into the response of gastrointestinal disorders to probiotics, with a focus on strain-specific efficacy. By excluding animal studies, our synthesis solely focused on the effects of the intervention on human participants, which enhance the validity and reliability of our article. However, despite its well-designed methodology, further research is required to gain a more comprehensive understanding of the topic.

### Clinical implications

The findings of this study suggest that both single-organism and composite probiotic formulations are effective complementary therapies for treating various gastrointestinal disorders in pediatric patients aged 18 years and under. As such, healthcare professionals should consider incorporating probiotics into standard treatment regimens. Gastrointestinal disorders among children are a significant cause of mortality, and probiotics have proven to be a safe and effective intervention. Fortunately, The National Institute of Health (NIH) has progressed with multiple ongoing clinical trials that aim to cater to various gastrointestinal disorders. A list of ongoing clinical trials listed in Table 5 provide valuable resources for clinicians and researchers interested in studying the effectiveness of strain-specific probiotic formulations for various gastrointestinal disorders in different age groups. However, there are many challenges to there are some challenges in clinical implications. The American Academy of Pediatrics 2021 guidelines highlight several warnings against recommending probiotics for preterm infants, particularly in infants with extremely low birth weight, including the absence of positive outcomes in a large RCT from the UK, the shortages of pharmaceutical-grade probiotics in the USA, the diversity of probiotic strains, demographic baselines of participants, and setting, and insufficient safety data^80^. Probiotic usage in healthy individuals may be safe, but in early newborns, it has been linked to an increased likelihood of infection and/or morbidity^81^ and underweight newborns^82^. This is most likely attributed to the transmigration of the administered strain or strains over the intestinal wall is most likely involved in the pathophysiology of probiotic sepsis^83^. It may further be challenging to detect because of difficulty forming colonies of obligate anaerobes using conventional culture techniques^84^.

**Table 5:** List of ongoing trials analyzing single-organism and composite probiotic formulations against gastrointestinal diseases.

Single-organism probiotic vs. placebo		
Trial ID	Age	Intervention
NCT04160767^69^	up to 14 years	Drug: Probiotic Vivomixx Behavioral: Gluten-free diet Other: Placebo
NCT03562221^70^	4 months to 4 months	Other: Gluten-free diet Dietary Supplement: Probiotics Dietary Supplement: Placebo
NCT04103216^71^	12 months to 36 months	Nitazoxanide with Lactobacillus Reuteri DSM 17938 Nitazoxanide
Composite Probiotics vs. Placebo		
NCT04922476^72^	8 years to 18 years	Dietary Supplement: Alflorex
NCT04021303^73^	4 to 12 months old	Dietary Supplement: Experimental cereal Dietary Supplement: Conventional cereal
NCT04541771^74^	28 weeks to 34 weeks	Drug: Lactobacillus Reuteri DSM 17938 Drug: Placebo
NCT04014660^75^	10 years to 18 years	Probiotic L.plantarum Heal 9 and L.paracasei 8700:2

Probiotics can also lead to harmful metabolic activities. Increased D-lactate, which may result in D-lactate acidosis, is one example. Not only do the majority of preterm babies already likely to be acidotic, but blood gases cannot regularly quantify D-lactate, which makes it extremely challenging to catch^84^ Probiotics also possess the potential to sometimes cause allergic responses, especially when Saccharomyces boulardii is employed in those who have a history of yeast allergies. In the early stages of treatment, abdominal pain and bloating are possible side effects. Antibiotic resistance genes, like those of enterococci, can also be transferred on by some probiotics. Some probiotic strains like Bacillus cereus may also release emetic and enterotoxin^85^. As such, a personalized plan is essential, and medical professionals should evaluate the unique condition and treatment response of every child. Selecting reliable brands and products is crucial since there may be variations in the quality and purity of probiotics. Since probiotics are typically sold as nutritional supplements rather than pharmaceuticals, the market for them is unregulated, allowing producers to alter the composition of their products or the method of manufacturing without appropriately addressing these concerns^86^. For instance, it was reported that contamination of a composite prebiotic was linked to a deadly case of gastrointestinal mucomycosis^87^. To relay trial results to clinical practise, it is crucial to ensure accurate product identity at the strain level both during research and throughout real clinical application^88^.

### Future insights and recommendations

It is recommended that guardians of pediatric age groups must be educated on the management of symptoms of gastrointestinal disorders. Oral rehydration for diarrhea, laxatives for constipation, and anti-emetics for vomiting are baseline treatments, this information can be provided to guardians through their doctor visits, health-care helpline and even educational health campaigns. The guardians must also ensure to keenly observe their children’s dietary and hygiene habits as that immensely affects gut health, routine checkup on bowel habits is recommended.

While the present study is a valuable addition to current knowledge on probiotics and their efficacy in treating gastrointestinal disorders, further research into identifying the optimal type, dose, and duration of probiotic therapy for specific conditions and strains that are most effective is needed to gain a more comprehensive understanding of their effectiveness. Specifically, future studies should focus on directly comparing single-organism and composite probiotic formulations, as well as examining the specific strains used as interventions. This would provide more precise guidelines for selecting effective probiotic formulations. Preclinical studies on mice have shown clinical benefits in alleviating symptoms following gastrointestinal diseases^69,89^. However, the effects observed in animal models do not necessarily translate to humans^90^, and further research is necessary to identify the molecular players involved including the gut-brain axis. Furthermore, despite the promising results, caution is necessary when using probiotics in the treatment of NEC. The treatment’s effectiveness depends on factors such as the specific strains used, the dosage, the mode of administration, and the inclusion of prebiotics. The patient’s characteristics, such as their baseline risk concerning birth weight, environmental exposure to microorganisms, and diet, should also be taken into account^58^. The long-term effects of using probiotics on the natural gut microbiome and their impact on gut health need more research. Furthermore, large-scale studies analyzing microbiota signatures related to various disorders, like celiac disease, can help clinicians choose the most effective probiotic formulations for functional gastrointestinal disorders in children.

### Limitations

One limitation of this study is the relatively small sample size and demographic scope, which may not be representative of a broader population or geographical area. Unfortunately, for the majority of the aforementioned clinical indications, there are also studies of similarly high methodological quality featuring opposing and negative results which cause disparity in overall inferences. In addition, the techniques employed in these experiments vary greatly, and encompass estimations from cell cultures, in vitro studies animal models, and human research. The diversity of strains investigated in probiotics research is another factor contributing to its unpredictability. Even now, Lactobacillus and Bifidobacterium genera, Lactococcus species, Streptococcus thermophilus, E. coli, and Saccharomyces boulardii are the predominant microorganisms employed in the probiotics sector. Certain health-related mechanisms of action—like the synthesis of bile salt hydrolases—are shared by several probiotic genera and species, while other characteristics may be unique to a particular strain or they might need to interact with one another to have an outcome. In addition, human responses to the same intervention may vary due to their great degree of heterogeneity in terms of nutrition, age range, genetic makeup, and gut microbiome composition, which sets them apart from animal models. It is also important to consider that individual responses to probiotics for gastrointestinal disorders can vary based on metabolites, genetics, and environmental factors. Therefore, the results obtained in this study may not be generalizable to all populations Lastly, a large number of probiotic research are financed by probiotic industry commercial entities or by professional advocacy groups closely affiliated and supported by the same industry. Additionally, biased results may occur due to the lack of randomization in the sample selection process. There is a need for independent verification of effectiveness claims through nonbiased research. Future human-based trials should also aim to overcome these limitations by conducting larger and more diverse studies, and implementing rigorous randomization protocols to reduce potential sources of bias scientific and medical organizations.

## Conclusion

In conclusion, this study highlights the potential of both single-organism and composite probiotic formulations as effective complementary therapies for gastrointestinal disorders in pediatric patients. However, due to inconsistency and limitation of our paper we cannot conclude which type of formulation, single or composite, is superior over the other. Hence, this paper warrants large-scale trials to validate the efficacy of either type. Overall, healthcare professionals should consider incorporating probiotics into standard treatment regimens and educate guardians on symptom management and gut health. Addressing colonization resistance is crucial for optimizing probiotic therapy, and the development of predictive algorithms based on host and microbiome features can aid in personalized treatment strategies. Further research into various gut microbes and microbiomes with specific demographics is recommended to enhance our understanding and application of probiotic interventions.

## Ethical approval

Not applicable.

## Consent

Not applicable.

## Sources of funding

The author(s) received no financial support for the research, authorship, and/or publication of this article.

## Author contribution

A.S.: conceptualized the original draft; A.S., S.I.A., and R.H.: visualized; A.S., R.H., S.I.A., L.I.V., K.Q., N.F., A.A., V.P., S.O., and M.A.H.: contributed to writing the original draft; A.S., S.O., M.A.H.: reviewed the final draft.

## Conflicts of interest disclosures

The author(s) declared no potential conflicts of interest concerning the research, authorship, and/or publication of this article.

## Research registration unique identifying number (UIN)

1. Name of the registry: not applicable.
2. Unique identifying number or registration ID: not applicable.
3. Hyperlink to your specific registration (must be publicly accessible and will be checked): not applicable.

## Guarantor

All authors accept full responsibility for the work and/or the conduct of the study, had access to the data, and controlled the decision to publish.

## Data availability statement

Available upon reasonable request from corresponding author.

## Provenance and peer review

Not commissioned, externally peer-reviewed.
